# Editorial: In celebration of women in science: Lipids, membranes, and membranous organelles

**DOI:** 10.3389/fmolb.2023.1187356

**Published:** 2023-04-13

**Authors:** Isabel M. López-Lara, Elena G. Govorunova

**Affiliations:** ^1^ Centro de Ciencias Genómicas, Universidad Nacional Autónoma de México, Cuernavaca, Mexico; ^2^ Department of Biochemistry and Molecular Biology, Center for Membrane Biology, The University of Texas Health Science Center at Houston McGovern Medical School, Houston, TX, United States

**Keywords:** women in science, membrane lipid, membrane transport protein, fatty acid, lipid bilayer, lipid remodeling

Integral membrane proteins have always been the focus of biomedical research because of their central role in transmembrane transport and signaling, energy transduction, maintaining of homeostasis, and numerous other cellular processes (Sachs and Engelman, 2006). In contrast, a lipid bilayer was initially considered as a relatively inert background for protein players. However, experimental evidence of functional modulation of membrane proteins by lipids challenged this view already ∼30 years ago (Bogdanov and Dowhan, 1995). Since then, a bewildering complexity has been revealed. Protein function is controlled, on the one hand, by bulk biophysical properties of the bilayer, such as its thickness, fluidity and elastic stress (Baccouch et al., 2022), and, on the other hand, by specific lipid-protein interactions (Jodaitis et al., 2021).

This Research Topic on the Celebration of Women in Science features articles by women authors and principal investigators active in the fields of Lipids, Membranes, and Membraneous Organelles. Frontiers in Molecular Biosciences has launched the Specialty Section “Lipids, Membranes, and Membranous Organelles” to create a forum for the exchange of results and ideas on the structure and function of membrane lipids and proteins, and on the entire membrane as a dynamic system. A dynamic view of the membrane reveals previously unrecognized regulatory mechanisms and facilitates understanding of cellular pathologies resulting from disruption of the normal membrane structure and function. This Research Topic is an inaugural Research Topic for this new Specialty Section and provides a snapshot of research conducted by women in the field. Contributors to this Research Topic examine various aspects of lipid, protein and membrane complexity using a wide range of approaches, from classical biochemistry (Stępniak et al.) to electron paramagnetic resonance (EPR) spectroscopy (Bartucci and Aloi), molecular modeling (Hryc et al.), confocal microscopy (Bulatova et al.) and lipidomic analyses (Balbi et al.).

A research team led by Malgorzata Karbownik-Lewinska (Stępniak et al.) found that membrane lipids in the thyroid comparing to non-endocrine tissues are less sensitive to pro-oxidative effects of Fenton reaction substrates, whereas melatonin decreased lipid peroxidation regardless of the oxidative stress associated with thyroid hormone production.

A mini-review by Bartucci and Aloi summarizes recent advances in probing librational (swaying) dynamics of lipids in model and natural membranes by electron spin echo (ESE) methods of time-resolved, pulsed EPR spectroscopy at cryogenic temperatures.

In plants, fatty acids (FAs) are synthesized in the chloroplast stroma and transferred to the endoplasmic reticulum (ER), where they are assembled into acyl lipids, including triacylglycerol stored in the oil bodies. In a collaborative paper by the groups of Katrin Philippar, Yonghua Li-Beisson and Michael Schroda (Peter et al.), the authors characterized FAX (fatty acid export) proteins in the unicellular green alga *Chlamydomonas reinhardtii* used for biofuel and biomaterial production.

P-glycoprotein (Pgp) is a multidrug transporter that binds a wide variety of hydrophobic compounds including anticancer drugs and uses the energy of ATP to pump the drugs out of the cells. The research group of Ina L. Urbatsch systematically investigated modulation of verapamil-stimulated ATPase activity of Pgp by artificial lipid mixtures that mimic the lipid composition of mammalian plasma membranes (Tran et al.).

Hyrc et al. carried out computational studies to identify the network of interactions of the lipid/water interfase of the thylakoid-forming lipids monogalactosyldiacylglycerol (MGDG) and digalactosyldiacylglycerol (DGDG). They concluded that the interaction network at the DGDG bilayer interface is more stable than in the MGDG bilayer.

The membrane protein NaPi2b is a sodium-dependent phosphate transporter that has been demonstrated to be overexpressed in ovarian and other cancers. A research team led by Ramziya Kiyamova and Mikhail Bogdanov (Bulatova et al.) show experimental evidence of the topology of untagged NaPi2b transporter in intact ovarian cancer cells. This precise information is of great importance for engineering therapeutic antibodies directed to fully accessible extramembrane domains.

A proper lipid composition of the membrane is essential for all forms of life. In humans, mutation of the *TAFAZZIN* gene leads to Barth syndrome, a mitochondrial lipid disorder causing severe cardiomyopathy. TAFAZZIN is a transacylase that is required for remodeling of cardiolipin acyl chains. Liang et al. present a perspective that focus on the link between TAFAZZIN dysfunction and an elevated level of reactive oxygen species (ROS). Authors propose that understanding the mechanism that leads to ROS formation in Barth syndrome can provide new tools for treatment.

Some microbial organisms are faced with harsh environmental conditions and to maintain membrane integrity they use different strategies. Tamby et al. review membrane lipid adaptations used by microbes living in the extreme conditions of the deep-see that is characterized by high hydrostatic pressure and low temperatures. To maintain fluidity of the membranes, bacteria increase unsaturation of fatty acyl chains while archaea show a rise in cyclisation of alkyl chains.

The lipid bilayer constitutes a semipermeable barrier for exogenous substances, and it is also the target for some molecules, among them membrane-acting antibiotics like daptomycin (Nguyen et al.) or the aegerolysin-based protein complexes derived from *Pleurotus* (Balbi et al.). Nguyen et al. review the mechanisms adopted by the bacterial cell membrane of Gram-positive pathogens acquiring resistance to the antibiotic daptomycin. Generally, changes in phospholipids are responsible for the acquired resistance to daptomycin.

Protein complexes of aegerolysins cause pores in insect gut cells by binding specifically to the sphingolipid ceramide phosphoethanolamine (CPE). Balbi et al. present evidence that also the sphingolipid ceramide aminoethylphosphonate (CAEP) can act as high-affinity receptor for aegerolysins protein complexes. CAEP is specifically found in plant pathogen oomycetes and in fouling marine invertebrates, and aegerolysin protein complexes could be used to eliminate them.

To conclude, we would like to note that the current view attributes underrepresentation of women in science, technology, engineering, and mathematics (STEM) to a complex interplay of social and cultural forces (Girelli, 2022). According to the United States Bureau of Labor Statistics, the gender gap is particularly large in some of the fastest-growing and highest-paid jobs such as computer programmers (only 19.5% of them were women in the year 2021). However, in biological sciences women were represented much better at 48.1% in the same year. This Research Topic is, no doubt, yet another demonstration of the success of female investigators in science.
